# Traumatic experiences, alexithymia, and posttraumatic symptomatology: a cross-sectional population-based study in Germany

**DOI:** 10.3402/ejpt.v5.23870

**Published:** 2014-08-27

**Authors:** Svenja Eichhorn, Elmar Brähler, Matthias Franz, Michael Friedrich, Heide Glaesmer

**Affiliations:** 1 Department of Medical Psychology and Medical Sociology, University of Leipzig, Leipzig, Germany; 2 Department of Psychosomatic Medicine and Psychotherapy, University Medical Center Mainz, Mainz, Germany; 3 Clinical Institute for Psychosomatic Medicine and Psychotherapy, Medical Faculty, Heinrich-Heine-University Düsseldorf, Düsseldorf, Germany

**Keywords:** PTSD, TAS-20, multiple and complex traumatization, avoidance/numbing, mediation

## Abstract

**Objective:**

Previous studies have established an association between number of traumatic experiences and alexithymia. The present study examines this relationship in a large-scale representative sample of the German general population (*N*=2,507) and explores the potential mediating effects of posttraumatic symptomatology, particularly *avoidance/numbing*.

**Methods:**

Alexithymia was assessed with the German version of the Toronto Alexithymia Scale (TAS-20). Posttraumatic symptomatology was operationalized by the symptom score of the modified German version of the Posttraumatic Symptom Scale, and traumatic experiences were assessed with the trauma list of the Munich Composite International Diagnostic Interview. Two mediation analyses were conducted.

**Results:**

Of the total sample, 24.2% (*n*=606) reported at least one traumatic experience, 10.6% (*n*=258) were classified as alexithymic, and 2.4% (*n*=59) fulfilled the criteria of posttraumatic stress disorder (PTSD). Participants who had survived five or more traumatic experiences had significantly higher alexithymia sum scores. The PTSD symptom cluster *avoidance/numbing* mediated the association between the number of traumatic experiences and alexithymia.

**Conclusions:**

Our findings illustrate an association between number of traumatic experiences and alexithymia and the influence of emotional avoidance and numbing within this relationship. The significant relationship between alexithymia and number of traumatic experiences in a general population sample further supports the concept of multiple and complex traumatization as associated with alexithymia. The results suggest the importance of further investigations determining the causal impact of alexithymia both as a potential premorbid trait and as consequence of traumatization. Lastly, future investigations are needed to clarify alexithymia as a distinct trauma-relevant characteristic for better diagnostics and specialized trauma-integrative therapy.

Alexithymia is defined as the limited ability to identify and describe feelings, including external-oriented thinking (Bagby, Parker, & Taylor, 1994a; Bagby, Taylor, & Parker, 1994b). Alexithymia has been investigated in various populations over the last several years. Etiological theories have identified alexithymia as a consequence of environmental influences (Gundel, Ceballos-Baumann, & Von Rad, 2002; Taylor, Bagby, & Parker, 1997) and have, in addition, suggested a genetic pathway for developing alexithymia (Jorgensen et al., 2002). Taylor and Bagby recently presented an overview of alexithymia in the context of psychoanalysis and empirical research (Taylor & Bagby, 2013). The impact of alexithymia and an external-oriented thinking on anxiety patients’ motivation for seeking psychosocial treatment was reported by Rufer, Moergeli, Moritz, Drabe, and Weidt (2014).

Traumatization has often been investigated as an environmental factor for alexithymia. To date, traumatization, occurring both in childhood and in adulthood, has been identified as the most important known risk factor for developing alexithymia. Posttraumatic stress symptoms and posttraumatic stress disorder (PTSD) are therefore frequently discussed factors connecting traumatic experiences and alexithymia (Declercq, Vanheule, & Deheegher, 2010; Fukunishi, Sasaki, Chishima, Anze, & Saijo, 1996; McCaslin et al., 2006; Sondergaard & Theorell, 2004; Spitzer et al., 2013; Spitzer, Vogel, Barnow, Freyberger, & Grabe, 2007; Zlotnick, Mattia, & Zimmerman, 2001). So far, this relationship has been poorly investigated in population-based studies. Yehuda et al. found a positive association between PTSD and alexithymia in Holocaust survivors, but no association between traumatic experiences and alexithymia. Because, according to their results, alexithymia seems more connected to posttraumatic symptoms than to trauma exposure itself, they conclude that alexithymia might be a preexisting condition that increases the probability of developing PTSD (Yehuda et al., 1997). Other studies showed that the inability to identify one's own feelings is highly congruent with the construct of PTSD (Evren, Dalbudak, Cetin, Durkaya, & Evren, 2010; Sondergaard & Theorell, 2004). Several studies indicate a phenomenological overlap between emotional numbing as a symptom of PTSD and alexithymia, which is also characterized by disordered affect regulation (Badura, 2003; Declercq et al., 2010; Fukunishi et al., 1996). Frewen, Dozois, Neufeld, and Lanius (2008) point out that the type of trauma experienced is an important factor influencing the probability of developing alexithymia. Complex or multiple traumatization is thought to increase the risk of alexithymia (Cloitre, Scarvalone, & Difede, 1997).

In conclusion, alexithymia is, on the one hand, often considered a premorbid trait that facilitates the development of PTSD as a response to traumatization and a risk factor for a chronic course of PTSD. On the other hand, there has been theoretical discussion, suggesting that alexithymia might be a component of PTSD and therefore one of the consequences of traumatic experiences. The purpose of the current study was to evaluate this idea in a population-based study.

The prevalence of traumatic experiences and PTSD within the general population has been examined internationally in several studies (Breslau, 2009; Kessler, 2005; Kessler, Kammerer, Hoffmann, & Traue, 2010; Van Ameringen, Mancini, Patterson, & Boyle, 2008). In Germany, the percentage of people in the population who have had at least one traumatic experience ranges from 24 to 55% (Hauffa et al., 2011; Maercker, Forstmeier, Wagner, Glaesmer, & Brähler, 2008; Spitzer et al., 2008), much lower than the 90% or more rate reported in US studies (Breslau, 2009). One-month-prevalence of PTSD in the German population is comparable to Canadian findings with rates between 2 and 3% (Maercker et al., 2008; Van Ameringen et al., 2008).

Very few population-based studies have focused on alexithymia. Those that do exist indicate prevalence rates ranging from 10% in the German population (Franz et al., 2008) to 26% in a Finnish study (Tolmunen et al., 2011). The present work constitutes the first international population-based study (*N=*2,507) examining the association between number of traumatic experiences, alexithymia, and PTSD symptomatology. We were especially interested in the mediating effect of the PTSD symptom cluster *avoidance/numbing* on this association.

After reporting alexithymia and PTSD prevalences and means, the following hypotheses were tested:

1. People with a higher number of traumatic experiences will show increased alexithymia manifestation.
2. Posttraumatic symptomatology will have a mediating effect on the association between the number of traumatic experiences and alexithymia.
3. The PTSD symptom cluster *avoidance/numbing* is the main mediating factor affecting the association between number of traumatic experiences and alexithymia.

It should be noted that the data are cross-sectional in nature and do not support a causal analysis.

Figure 1 illustrates total (path c), direct (path c′), and indirect effects (path c minus path c′) (Modell A and B) between the variables tested.

**Fig. 1:**
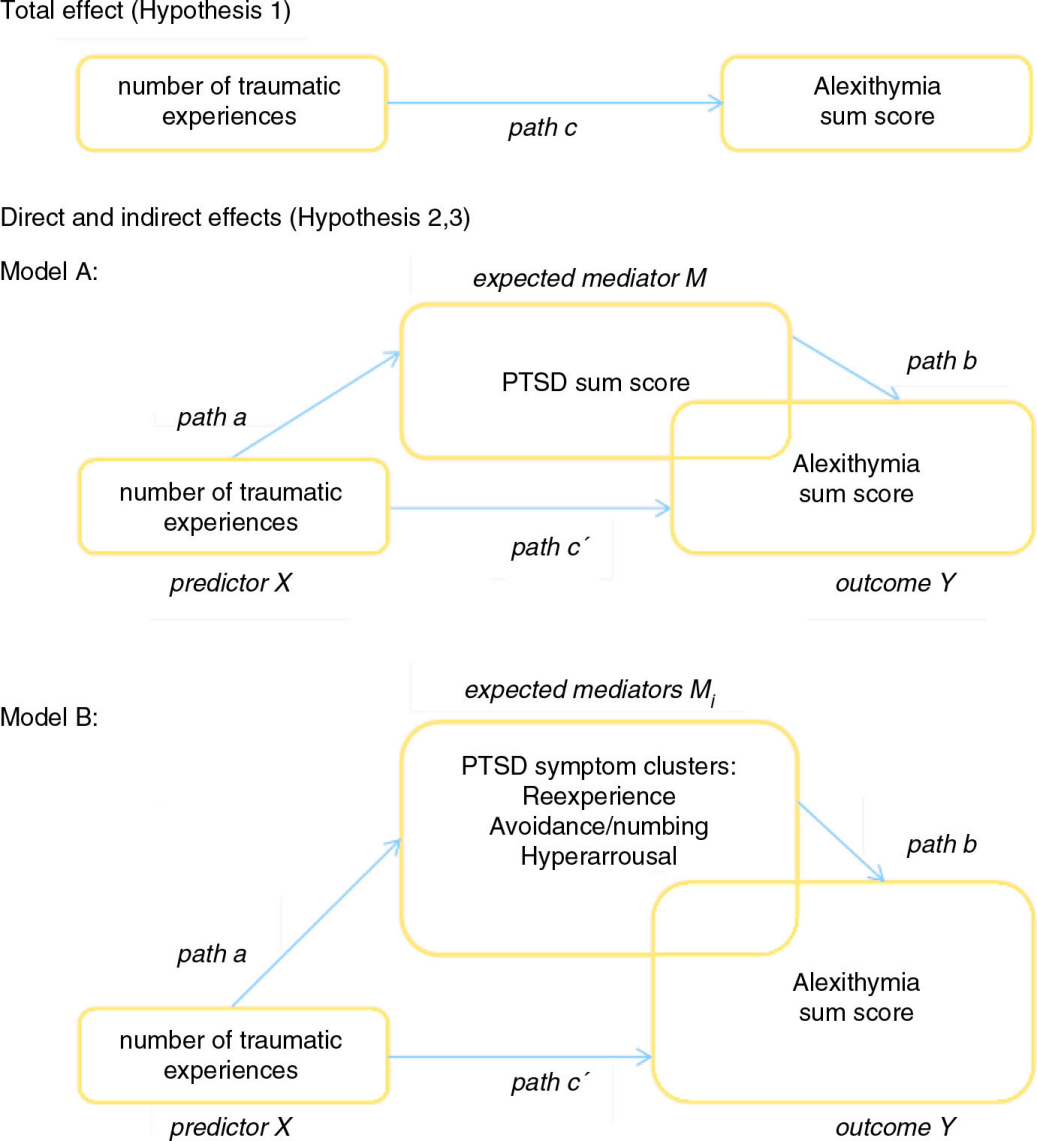
Tested models of total, direct, and indirect effects.

To report a mediation-effect, the following requirements have to be fulfilled (Frazier, Tix, & Barron, 2004):

1. Significant relation between predictor and outcome (see path c, Fig. 1)
2. Significant relation between predictor and mediator (see path a, Fig. 1)
3. Significant relation between mediator and outcome (see path b, Fig. 1), if controlled for the predictor's effect on the outcome
4. The strength of the relation between predictor and outcome is significantly reduced when the mediator is added to the model (see path c′, Fig. 1/to be tested with the Sobel test)

## Methods

### Participants and study protocol

A representative sample of the German general population was selected with the assistance of a demographic consulting company (USUMA, Berlin, Germany). The total area of Germany was separated into 258 sample areas representing the different regions of the country. Households of the respective area and members of these households fulfilling the inclusion criteria (age 14 or above, able to read and understand the German language) were selected randomly. The sample is representative in terms of age and sex. A first attempt to establish contact was made at 4,243 addresses, of which 4,118 were valid. If a candidate could not be reached on the first try, a maximum of three additional attempts were made to contact them. Participants were visited by study assistants, informed about the investigation, and given self-rating questionnaires. A total of 2,549 people agreed to participate and completed the self-rating questionnaires in July and August of 2005 (participation rate: 61.9%). The assistants waited until participants answered all questionnaires, and offered help if the meaning of questions was not clear. After elimination of 42 interview-sets due to missing data, 2,507 (60.9%) participants were included in the analysis (*M*: 49.2 years; SD: 17.9 years). The survey met the ethical guidelines of the international code of marketing and social research practice by the international chamber of commerce and the European society of opinion and marketing research.

To test the impact of posttraumatic symptomatology on the association between number of traumatic experiences and alexithymia, two mediation analyses were conducted (Model A and Model B) on a subsample of participants who had either experienced at least one traumatic event (*n*=606) or fulfilled A1-criterion according to the DSM-VI-TR (American Psychiatric Association, 2000). After eliminating missing values, the analyses included *n*=468 participants. Table 1 gives an overview of the demographic characteristics of the full study sample (*N*=2,507) and the subsample with trauma experience (*n=*606). The most important difference between the full study sample and the subsample is age, most likely because of the preconditioned A1-trauma criterion. This means the subsample contains more elderly participants, who lived through World War II and were thus more likely to have experienced traumas associated with the exceptional nature of that situation, including among others, higher than average rates of widowhood.

**Table 1:** Sociodemographic characteristics

	*N=*2,507	%	*N=*606	%	*χ* ^2^	*φ*
Sex				.029		
Male	1,155	46.1	281	46.4		
Female	1,352	53.9	325	53.6		
Age					413.620**	.41
14–40 years	862	34.4	93	15.3		
41–60 years	867	34.6	136	22.4		
61–75 years	598	23.9	256	42.2		
Older than 75 years	180	7.2	121	20.0		
Partnership status					201.338**	.28
Married/living together	1,375	54.8	312	51.5		
Married/not living together	31	1.2	8	1.3		
Single	551	22.0	67	11.1		
Divorced	234	9.3	48	7.9		
Widowed	316	12.6	171	28.2		
School graduation					70.871**	.17
Without graduation/pupil	119	4.7	24	4.0		
Secondary graduation	2,055	82.0	500	82.5		
A-form	158	6.3	32	5.3		
University/technical college	175	7.0	50	8.3		

** *p*≤.01.

### Instruments

Corresponding to the trauma list of the PTSD module of the Munich Composite International Diagnostic Interview (M-CIDI) (Wittchen & Pfister, 1997), eight potential traumatizing events were given (e.g., “you were the victim of rape”; “… of a natural disaster”; “… you received serious bodily threats (as with a weapon), were attacked, injured, or tortured”; “… you had horrible experiences during war service”), and an open question about “another terrible event or catastrophe”. Additionally, an inquiry was made concerning three war-related events (“You were bombed out”; “You were driven out of your homeland”; “You had awful experiences during war effort”). Participants had to answer the trauma items with “yes” or “no”.

### Modified PTSD Symptom Scale

PTSD according to DSM-IV was assessed with the German modified and shortened version of the Posttraumatic Symptom Scale (PSS) by Foa and colleagues (Breslau, Peterson, Kessler, & Schultz, 1999; Foa, Riggs, Dancu, & Rothbaum, 1993; Maercker et al., 2008; Stieglitz, Frommberger, Foa, & Berger, 2001), analogue to Maercker et al. (2008). The answers refer to the occurrence of symptoms in the last month on a four-point scale from 0 (“not at all”), 1 (“once a week or more seldom”), 2 (“2–4 times per week/half the time”), to 3 (“several times per week/almost always”). PTSD was determined according to the DSM-IV (with the A1 and A2 criteria, B criteria and at least 4 of 7 symptoms according to Breslau with scale values ≥2 and F criteria) (Maercker et al., 2008).

The PSS by Breslau et al. (1999) has shown satisfactory reliability and validity in both American (Foa et al., 1993) and German studies (Stieglitz et al., 2001). The abbreviated item selection from Breslau et al., which turned out to be the most effective item selection according to receiver operating characteristic analysis, defined positive PTSD cases compared to the complete symptom criteria list with 80% sensitivity, a specificity of 97%, a positive predictive value of 71%, and a negative predictive value of 98% (Breslau et al., 1999). The German version of the PSS shows similar psychometric properties to those reported by Foa et al. (1993). In our study, the modified scale shows very good internal consistency (Cronbach's *α*=.93). The impairment criterion was excluded from PTSD symptom score calculations because of its single-item character.

### Toronto Alexithymia Scale

The German version (Franz, Schneider, & Schafer, 2001; Franz, Schneider, Schafer, Schmitz, & Zweyer, 2001; Popp et al., 2008) of Bagby's 20-item Toronto Alexithymia Scale (TAS-20) (Bagby et al., 1994a, b) was used to assess alexithymia. The scale's subscales measure three dimensions of the construct of alexithymia: “difficulty identifying feelings”, “difficulty describing feelings”, and “external-oriented thinking.” Each of the 20 items has to be answered on a five-point likert-scale (“not at all” to “fully applicable”). The TAS-20 sum score is calculated by adding up all item scores. “High alexithymics” were defined as the subgroup above the 90th percentile of the distribution, according to Franz et al. (2008). Both sum scores and cut-off scores were used in the computations. In our study, the TAS-20 showed good internal consistency (Cronbach's *α*=.80).

### Statistical analyses

The difference in alexithymia sum scores between the categories of traumatic experiences was assessed with univariate analysis of variance (ANOVA) while post-hoc p-values were Bonferroni adjusted. Sociodemographic differences between the full and subsample as well as sex and age effects on alexithymia and PTSD symptomatology were tested with *χ* ^2^—tests and univariate ANOVA. The postulated mediating effect of PTSD symptomatology within the association of traumatic experiences and alexithymia was examined by two mediation analyses. The hypotheses were analyzed stepwise with regression analyses implemented by macro for SPSS written by Hayes (2012). The calculations were run with bootstrapped estimates (Preacher & Hayes, 2008). The analyses included a Sobel test to test if the indirect effects of the mediating variables are significant (Sobel, 1986). Sex and age were included as possible confounders. The analyses were conducted with IBM SPSS Statistics version 20 for Windows.

## Results

### Lifetime prevalence of trauma by sex and age

Table 2 gives an overview of the prevalence of the traumatic experiences in the total sample. Twenty-four percent of the sample reported at least one traumatic experience, 14.4% reported at least one war-related trauma, and 10.9% reported at least one civilian traumatic experience.

**Table 2:** Traumatic experiences (*N=*2,507)

	*n*	%
War related (at least one)	360	14.4
War effort/combat action	206	8.2
Bombing	177	7.1
Displacement/eviction	171	6.8
Civilian (at least one)	273	10.9
Rape	18	0.7
Childhood abuse	29	1.2
Prisoner/hostage	40	1.6
Serious accident	112	4.5
Physical violence	98	3.9
Life-threatening illness	73	2.9
Natural catastrophe	20	0.8
Witness of trauma	214	8.5
Other	90	3.6
Number of traumatic experiences (total: *M=*0.5; SD=1.114)		
0	1,847	75.8
1	287	11.8
2–4	259	10.6
5–8	44	1.8
total	2,437	100

### Alexithymia, PTSD, and posttraumatic symptomatology

**Fig. 2:**
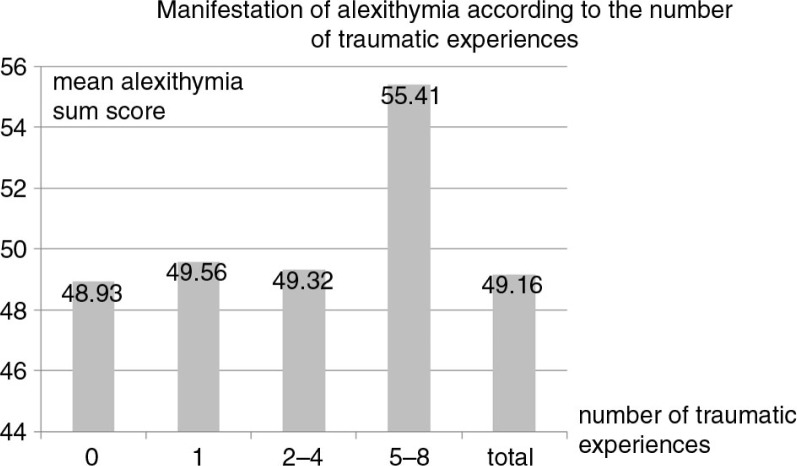
Alexithymia manifestation according to the number of traumatic experiences.

Figure 2 illustrates the mean alexithymia sum scores of the four categories of traumatization as well as test statistics of univariate ANOVA. We found a significant difference in alexithymia manifestation between the trauma categories (*F*(3, 2433)*=*7.388; *p*≤.001; *η* ^2^=.01). Alexithymia sum scores were significantly lower for those participants with none to four traumatic experiences (*M=*49.1; SD=9.175) as compared to those with five to eight traumatic experiences (*M =*55.4; SD=9.834) (*F*(1, 2435)*=*20.720; *p*≤.001; *η* ^2^=.01). Pairwise comparisons indicated that there was no significant difference between any groups with none (post-hoc mean difference: −5.845; *p*≤.001), one (post-hoc mean difference: −6.089; *p*≤.001), or two to four traumatic experiences (post-hoc mean difference: 6.089; *p*≤.001).

Table 3 shows the prevalence of PTSD and alexithymia by sex and age as well as means of posttraumatic symptomatology and alexithymia. A total of 10.6% of our sample was identified as “high alexithymic” (threshold=^90th percentile/threshold ≥61). There were no sex differences in the TAS-20 sum scores nor in the proportion of individuals categorized as “high” alexithymics.

**Table 3:** PTSD and alexithymia by sex and age

	Distribution by sex							Distribution by age									
		
	Total		Women		Men			14–40 years		41–60 years		61–75 years		>75 years			
					
	*N* (%)		*N* (%)		*N* (%)		*χ* ^2^	*N* (%)		*N* (%)		*N* (%)		*N* (%)		*χ* ^2^	*ϕ*
Alexithymia	*N=*2,437		*N=*1,309		*N=*1,128			*N=*834		*N=*846		*N=*582		*N=*175			
Cut off at 90th	258 (10.6)		127 (9.7)		131 (11.6)		2.338	106 (12.7)		77 (9.1)		61 (10.5)		14 (8.0)		7.186	.05
Percentile/threshold ≥61^a^																	
PTSD^b^ (in the entire	*N=*2,448		*N=*1,316		*N=*1,132			*N=*850		*N=*848		*N=*580		*N=*170			
sample)	59 (2.4)		36 (2.7)		23 (2.0)		1.222	12 (1.4)		19 (2.2)		18 (3.1)		10 (5.7)		12.719**	.07
PTSD (in those	*N=*606		*N=*325		*N=*281			*N=*93		*N=*136		*N=*256		*N=*121			
participants with trauma exposure)	59 (9.7)		36 (11.1)		23 (8.2)		.0290	12 (13.0)		19 (14.0)		18 (7.0)		10 (8.3)		413.620**	.10
	*M*	SD	*M*	SD	*M*	SD	*F*	*M*	SD	*M*	SD	*M*	SD	*M*	SD	*F*	*η* ^2^
PTSD symptomatology																	
Reexperiencing	2.07	0.98	2.07	0.98	2.06	0.97	.013	2.09	0.92	2.41	1.10	1.93	0.94	1.91	0.86	6.635**	.04
Avoidance/numbing	1.68	0.78	1.72	0.82	1.62	0.74	1.877	1.66	0.78	1.85	0.83	1.63	0.75	1.59	0.78	2.404	.02
Hyperarousal	1.84	0.93	1.90	0.97	1.77	0.87	2.424	1.84	0.98	2.05	0.99	1.74	0.89	1.80	0.87	2.619*	.02
Total	1.86	0.90	1.90	0.92	1.82	0.86	3.632	1.86	0.89	2.10	0.97	1.77	0.86	1.77	0.84	4.431**	.03
TAS-20 sum score	49.16	9.22	48.73	9.20	49.67	9.23	6.352	49.55	9.57	48.94	9.18	49.07	9.17	48.67	7.90	.850	.00

a According to Franz et al. (2008).

b According to Maercker et al. (2008).

** *p*≤.01

* *p*≤.05.

A total of 2.4% of the sample fit the criteria for full PTSD. Within the subsample (*n*=606), the prevalence of PTSD was approximately 10%. There was no sex difference in rates of PTSD (Table 3). The prevalence of PTSD increased significantly across the age groups from 1.4% (14–40 years) up to 5.7% (over 75 years). When considering only participants who mentioned at least one traumatic experience, a reverse effect can be observed (14–40 years: 13.0%; over 75 years: 8.3%) (see Table 3).

Considering the means of posttraumatic symptomatology, there are significant differences between the age groups in the total score as well as in the symptom clusters “*reexperiencing”* and “*hyperarousal”*.

### Mediation analyses: the association of traumatic experiences, posttraumatic symptomatology and alexithymia

Table 4 shows the results of the mediation analyses predicting alexithymia. To test mediating effects of posttraumatic symptomatology on this association, two mediation analyses were conducted (Model A with the mediator PTSD symptom score; Model B with the mediators *re-experience*, *avoidance/numbing*, and *hyperarousal*) (see Fig. 1).

**Table 4:** Testing mediation effects using linear regression

Model A (*N=*468)^a^					Model B (*N=*468)^a^				
Testing step 1 (total effect/path c)		*b*	*β* (SE)	*R* ^2^	Testing step 1 (total effect/path c)		*b*	*β* (SE)	*R* ^2^
Outcome	Alexithymia sum score				Outcome	Alexithymia sum score			
Confounder	Sex	1.111	.060 (.0473)		Confounder	Sex	1.111	.060 (.0473)	
	Age	−.049	−.094 (.0513)			Age	−.049	−.94 (.513)	
Predictor	Number of traumatic experiences	1.110***	.134*** (.0388)	.029	Predictor	Number of traumatic experiences	1.110***	.134*** (.0388)	.029
Testing step 2 (direct effect/path a)					Testing step 2.1 (direct effect/path a_1_)				
Outcome	PTSD sum score				Outcome	Re-experience			
Confounder	Sex	.082	.050 (.046)		Confounder	Sex	.010	.005 (.0458)	
	Age	−.007*	−.152* (.050)			Age	−.010***	−.175*** (.0497)	
Predictor	Number of traumatic experiences	.070**	.095** (.038)	.026	Predictor	Number of traumatic experiences	.085**	.097** (.0376)	.030
					Testing step 2.2 (direct effect/path a_2_)				
					Outcome	Avoidance/numbing			
					Confounder	Sex	.105	.067 (.0463)	
						Age	−.005*	−.24* (.0502)	
					Predictor	Number of traumatic experiences	.069**	.098** (.0379)	.024
					Testing step 2.3 (direct effect/path a_3_)				
					Outcome	Hyperarousal			
					Confounder	Sex	.130	.070 (.0462)	
						Age	−.006*	−.115* (.0502)	
					Predictor	Number of traumatic experiences	.057	.068 (.0379)	.018
Testing step 3 (direct, indirect effect/paths b and c′)					Testing step 3 (direct, indirect effect/paths b and c′)				
Outcome	Alexithymia sum score				Outcome	Alexithymia sum score			
Confounder	Sex	.775	.042 (.044)		Confounder	Sex	.666	.036 (.442)	
	Age	−.0198	−.038 (.049)			Age	−.022	−.043 (.0484)	
Mediator	PTSD sum score	4.124***	.099*** (.036)		Mediator	Re-experience	.436	.046 (.0740)	
Predictor	Number of traumatic experiences	.820** b	.099** (.037)	.153		Avoidance/numbing	3.827**	.325*** (.0778)	
						Hyperarousal	.294	.030 (.0742)	
					Predictor	Number of traumatic experiences	.793** b	.096** b (.0363)	.166

a Multiple Linear Regression Analysis with bootstrapped estimate (1,000 samples): subsample with trauma exposure, as required for reporting posttraumatic symptomatology.

b Significant indirect effect (reduction in regression coefficient *b*) (Sobel test);

*** *p*≤.001

** *p*≤.01

* *p*≤.05.

Because the total effect was significant (path c), the requirement for mediation step 1 was met. In step 2 (Model A), the relationship between the predictor and the mediator was tested. Because the regression coefficient *b* was significant, the condition for step 2 was met. The coefficient related to alexithymia and posttraumatic symptomatology (path b) was significant when controlling for the number of traumatic experiences (step 3). Step 3 also provides an estimate of path c′, the relation between the number of traumatic experiences and alexithymia, controlling for posttraumatic symptomatology. The coefficient of this relation was significant and significantly smaller than for path c, which was tested by a Sobel test. For Model A, there is an explained variance of 15% (see Hypothesis 2).

Model B includes the three symptom clusters of PTSD as mediators (*re-experience, avoidance/numbing, hyperarousal*). The same mediation steps were tested for this model as for Model A. Path c (step 1) is identical. That is why it has the same coefficient and was significant. The relations in path a were tested for each mediator (step 2_j_). Path a_1_ and path a_2_ were significant, proving the direct effect of the predictor (number of traumatic experiences) on both mediators. The requirements for mediation in step 2_3_ (path a_3_) were not met. A significant indirect effect was only shown for the mediator *avoidance/numbing* in step 3 (path b). The increase of the predictor's *b* coefficient (path c′) compared to the total effect (path c) was significant, as proven by a Sobel test. The posttraumatic symptom cluster *avoidance/numbing* remains as the only significant mediator in this analysis. Model B includes an explained variance of 17% (see Hypothesis 3).

Possible confounder variables (sex and age) were added to both models, whereas only age was significant in step 2 (Model A) and step 2_1–3_ (Model B). Increased age is associated with a lower total posttraumatic symptomatology or fewer reexperiencing, avoidance/numbing and hyperarousal symptoms, whereas the effect of the number of traumatic experiences remains significant in each analysis.

## Discussion

The present study examines the association between number of traumatic experiences and alexithymia within a large-scale representative sample of the German general population. A partially mediating effect of posttraumatic symptomatology, and the meaning of the PTSD symptom cluster *avoidance/numbing* in the context of alexithymia is demonstrated with two four-step mediation analyses.

Almost one-fourth of the sample reported having experienced at least one traumatic event in their lifetime. While this prevalence is much lower than those reported in studies from the United States (Breslau, 2009), it is in the lower range of the available findings from Germany (Hauffa et al., 2011; Spitzer et al., 2008). The most frequent traumatic experience reported was “war effort/combat action”, underlining the importance of traumatic experiences from World War II. The least frequent traumatic experience reported was “rape”, whereas we assume that this low prevalence results partly from a reporting bias due to taboo. A similarly low prevalence applies to PTSD, which is lower than reported in studies from the United States (Kessler, Sonnega, Bromet, Hughes, & Nelson, 1995) but in line with other population-based studies from Germany (Spitzer et al., 2008). Of the subgroup of people who had experienced at least one traumatic experience in their lifetime, the prevalence of PTSD is lower in the German elderly than in younger age groups. This finding might be attributable to a selection effect, insofar as most of the traumatic experiences go back to World War II. Because traumatic experiences are related to limited physical health and mortality (Glaesmer, Brähler, Gundel, & Riedel-Heller, 2011), many of the most severely traumatized people in older age brackets have either already died, were unable to participate, or live in nursing homes, etc. This found age effects for posttraumatic symptomatology did not appear for alexithymia.

Consistent with previous studies, our analyses indicate that alexithymia is associated with the number of traumatic experiences a person has undergone (Badura, 2003; Declercq et al., 2010; Frewen et al., 2008a; Fukunishi et al., 1996; Sondergaard & Theorell, 2004; Spitzer et al., 2007; Yehuda et al., 1997). Because of the high rates of alexithymia among those participants who had been repeatedly traumatized, the concept of multiple and complex traumatization as associated with alexithymia is supported by the analyses (see Hypothesis 1). The association between the number of traumatic experiences and alexithymia still remains, when the mediating effect of posttraumatic symptomatology is considered (see Hypothesis 2). Additionally, the importance of the symptom cluster *avoidance/numbing* was shown (see Hypothesis 3). This is in line with findings from several other studies (Badura, 2003; Declercq et al., 2010; Fukunishi et al., 1996), which have reported an association or a lack of independence between the PTSD symptom *numbing* and alexithymia.

When analyzing possible confounder variables, only age remains a significant negative predictor for posttraumatic symptomatology. This result is congruent with the earlier reported negative relation between PTSD prevalence and age across the lifespan and might demonstrate the discussed selection bias.

Given the results from this and other studies that alexithymia might be a consequence of multiple or complex traumatization (Frewen et al., 2008a; Sondergaard & Theorell, 2004; Zeitlin, Mcnally, & Cassiday, 1993) we consider that alexithymia might be then theoretically linked to another consequence of complex traumatization, complex PTSD or DESNOS (disorder of extreme stress, not otherwise specified (Ford, 1999; Van der Kolk & Courtois, 2005). Complex PTSD is proposed for listing in the upcoming WHO International Classification System of Diseases (ICD-11) as an independent clinical picture within the spectrum of trauma and stress-related disorders. We assume that complex PTSD symptomatology might be closer to alexithymia than simple PTSD symptomatology because of preceding multiple and/or long-term traumatization (Maercker, 2003) and the characterizing affect dysregulation (McLean, Toner, Jackson, Desrocher, & Stuckless, 2006). Cloitre and colleagues characterized complex PTSD as high in PTSD symptoms, as well as in affective and interpersonal problems and a negative self-concept (Cloitre, Garvert, Brewin, Bryant, & Maercker, 2013). Furthermore, people with symptoms of complex PTSD show a greater long-term functional impairment than those with simple PTSD (Cloitre et al., 2013). In this context, our findings underscore the necessity of future prospective research, which should focus on the type and extent of traumatization experienced by alexithymic or non-alexithymic people, with the aim of describing how alexithymia and traumatization are linked. If future longitudinal studies show alexithymia more clearly as a consequence of traumatization or multiple traumatization, specific trauma-integrative therapeutic approaches for individual settings should be developed.

Although our study has strengths such as a large sample size and a population-based approach, some factors limit the interpretation and generalizability of our findings. As mentioned in the introduction, the cross-sectional design of the study does not allow us to draw directional causal inferences concerning the association between posttraumatic symptomatology and alexithymia. Moreover, alexithymia as well as PTSD symptomatology were measured by self-report instruments. Consequentially, the validity of the results is reduced.

Traumatic experiences were assessed with the trauma list of the M-CIDI, which might be too restrictive and possibly lead to an underestimation of the prevalence of traumatic experiences. The divergence between the prevalence of traumatic experiences in different studies is at least partly attributable to different methodological approaches (e.g., type and number of traumatic experiences under study, open questions vs. list of traumatic experiences), and cultural and historical characteristics. Furthermore, the reported number of traumatic experiences does not allow conclusions about the relationships between types of traumatization and alexithymia. Future research should focus on specific types of traumatic experiences to explore their associations with alexithymia in more detail.

## Conclusions

Our study constitutes the first international population-based evidence for the mediating effect of posttraumatic symptomatology on the association between number of traumatic experiences and alexithymia. The strong association between alexithymia and posttraumatic symptomatology highlights the lack of conceptual and diagnostic consideration of alexithymia as it relates to PTSD (Badura, 2003) and complex PTSD formulations.
